# Indigenous practices of pregnant women at Dilokong hospital in Limpopo province, South Africa

**DOI:** 10.4102/curationis.v38i2.1553

**Published:** 2015-12-17

**Authors:** Mamagoro A. Mogawane, Tebogo M. Mothiba, Rambelani N. Malema

**Affiliations:** 1 Department of Nursing Science, University of Limpopo, South Africa

## Abstract

**Background:**

Indigenous practices (IPs) are experiences generated by people who are living in a specific regional context and cultural group. IPs are shaped by cultural traits that are passed from one generation to the next. IPs practices are rooted and embedded in society and, therefore, the practices become part of the people’s lifestyle. It is difficult to try and change these practices as people have adhered to them throughout their entire lives. The believe system plays a major role in health care seeking behaviour of individuals because they are informed by the IPs that are observed in their environment.

**Objectives:**

To explore and describe the IPs of pregnant women at Dilokong hospital in Limpopo province.

**Method:**

A qualitative, descriptive, explorative and contextual research design was used for the participants to describe the IPs used by pregnant women. Data were collected through unstructured one-on-one interviews.

**Results:**

The following four themes with sub-themes emerged from the data: IPs based on ancestral knowledge; IPs based on spiritual diviners versus church principles; restricted practices versus instructions followed during pregnancy; and labour and IPs during labour and delivery.

**Conclusion:**

IPs are regarded as an honourable health intervention by traditional health practitioners (THPs), families and pregnant women. IPs like cords around women’s waists are still observed during physical examinations. However, there is a reduction of prescribed indigenous oral medication used to accelerate labour because of their potential toxicity.

## Introduction and background

Indigenous practices (IPs) are expressed in songs, dances, beliefs, rituals, cultural values, myths, and the treatment of diseases by using herbs. IPs are still applied during pregnancy worldwide, for example, in Turkey and Africa where women’s reassurance depend on the local context and meaning of pregnancy. Evidence suggests the possibility that following those traditional practices during pregnancy has both therapeutic and harmful consequences (Ayaz & Efe 2008:285).

The study conducted by Gracey and King (2009:69) in Australia points out that almost 400 million indigenous people in the world have a low standard of health. Therefore, there is a need to acknowledge IPs by creating increased awareness campaigns, as well as political commitment and recognition of those serious and complex problems that are experienced by pregnant women who are using traditional medicine. The Department of Health (DoH) (2005:36) states that traditional health practitioners (THPs) are providing health care services in South Africa but their competencies are not recognised. Therefore, there is a need for the health care system to learn more about IPs in order to create a platform for the integration of services between Western medicine and IPs. In South Africa the THPs and indigenous birth attendance have been legalised under the *Traditional Health Practitioners Act*, Act 22 of 2007 (South Africa 2008:6). It is, therefore, imperative for midwives to understand the IPs of pregnant women. Mulaudzi and Ngomane (2003:23) indicate that 80% of black Africans depend on THPs for care because of poverty and inaccessibility to health facilities. The study conducted by Peltzer, Phaswana-Mafuya and Treger (2009:159) points out that THPs use IPs either to prevent or to heal childhood illness. With this background, the researcher was motivated to conduct a scientific research study about the indigenous practices (IPs) of pregnant women at the Dilokong hospital of the Greater Tubatse municipality in Limpopo province.

## Problem statement

It was evident during physical examination in January 2012 that 20 pregnant women who came for routine ante-natal care (ANC) were using IPs, as razor blade cuts were observed on their body, some were wearing robes made of animal skin around their abdomens, whereas some had smeared yellow egg yolk on the entire abdomen. Family members also brought them indigenous medication in cooldrink bottles and instructed them to drink which might be harmful to the unborn baby.

## Research aim

The aim of this study was to determine the IPs of pregnant women.

## Research objectives

To explore and describe the IPs of pregnant women at the Dilokong hospital in Limpopo province.

### Significance of the study

Knowledge about these indigenous practices could be used to develop guidelines for health care professionals regarding the advice they could give to pregnant women regarding IPs which could be harmful to the baby and the mother.

## Research methodology

### Design

A qualitative, explorative, descriptive, and contextual research design assisted the researcher to obtain complete and accurate information on IPs whilst providing care to pregnant women at the Dilokong hospital in the Greater Tubatse municipality of the Limpopo province. Brink, Van der Walt and Van Rensburg (2014:120) describe a qualitative research approach as a method to explore the meaning, and to describe and promote understanding, of the view of the participants in the context in which a particular action is taking place. Exploratory research is conducted to gain insight into the research phenomenon (Babbie & Mouton 2009:79; De Vos *et al*. 2006:144). Descriptive research presents specific details about a situation and social setting of the phenomenon studied (Babbie & Mouton 2009:271). Contextual interest by the researcher was aimed at understanding events of the phenomenon studied within the concrete, natural context of the participants in which the practice occurred; the pregnant women who were simultaneously making use of IPs whilst seeking health care at the Dilokong hospital in the Greater Tubatse municipality of Limpopo (Babbie & Mouton 2009:272). These pregnant women were between the ages of 18 to 38 years, 10 came from rural areas and 5 from semi-urban areas.

### Population and sampling

The target population was pregnant women who attended an ANC clinic of the Dilokong hospital in Limpopo during July and August 2013. A non-probability purposive sampling technique was used in this study. A purposive sample was achieved by including only the participants who had knowledge related to IPs during pregnancy. The participants included in the interview sessions were those who had evidence of razor blade cut marks, wearing robes around the abdomen, having being smeared with yellow egg yolk on the abdomen and also those who were found to be taking indigenous medication in cooldrink bottles. Data were collected from 15 participants until data saturation was reached (De Vos *et al.* 2006:328).

### Research setting

The study site was the Dilokong hospital which is a first level hospital that provides care for, amongst others, pregnant women. It is situated in the Greater Tubatse local municipality of the Sekhukhune district, Limpopo province in South Africa. The hospital serves patients from 14 primary health care clinics, 1 primary health care centre, 18 mobile clinics, 3 mine clinics and 1 gateway clinic.

### Data collection method

Unstructured one-on-one interviews (De Vos et al. 2007:292) were conducted during this study to determine the IPs of pregnant women at the Dilokong hospital. The following one central question was put in the same way to each participant:

’Will you kindly describe indigenous practices that are used during pregnancy that you know?’

The main question was followed by clarity-seeking questions to allow the participants to clarify areas where the researcher sought to increase and generate detailed data until saturation was reached (Brink, Van der Walt & Van Rensburg 2012:144). All interview sessions were recorded verbatim by using a voice recorder and field notes were written to capture non-verbal cues that were not captured by the voice recorder to supplement the data collected. Unstructured one-on-one interviews were conducted for a period of two months (July and August 2013) and the sessions lasted for approximately 30 to 40 minutes.

### Data analysis

Tesch’s inductive, descriptive coding technique (Creswell 2009:124) was used to analyse the data. The eight steps were used which included categorising, ordering, manipulating, summarising, and describing the data in meaningful terms. The final decision was then taken about the themes which emerged during data analysis. The themes and their sub-theme were arranged in columns based on their similarities. An independent coder was requested to analyse raw data. A meeting was held between the independent coder and the researcher to reach consensus about the categories they had identified independently.

## Ethical considerations

The ethical standards for nurse researchers as outlined by the Democratic Nursing Organisation of South Africa (DENOSA) (DENOSA 1998) were adhered to. Research ethical clearance was obtained from the Medunsa Research Ethics Committee (MREC). Permission for collecting data at the health facility was sought from the Limpopo Provincial Department of Health. Written informed consent was obtained voluntarily from each participant after the participants had been adequately given an outline of risks and benefits involved in the research project and before commencement of the interview sessions. To ensure confidentiality, participants were informed that the information that they provide during the interview sessions would not be revealed. No participants’ names were used in naming files created in the voice recorder (De Vos *et al*. 2006:84).

### Trustworthiness

The following strategies regarding credibility, dependability, confirmability and transferability, as described by Babbie and Mouton (2009:277) were adopted to ensure trustworthiness of data.

### Credibility

Credibility was ensured by extensive engagement with the participants in the field whilst collecting data during unstructured one-on-one interviews over a period of two months, until data saturation was reached (Babbie & Mouton 2009:277). All the interview field notes were sent to an independent coder and a consensus meeting was held in order to agree on codes that were identified independently.

### Dependability

The research methodology was described in detail to enhance the possibility of other researchers repeating the study.

### Confirmability

An inquiry auditor was used to check and assess whether the conclusions, findings, and interpretations were supported by collected data.

### Transferability

Transferability refers to the extent to which the findings could be applied in other contexts (Babbie & Mouton 2009:277) and it was ensured by purposive sampling of participants who had the knowledge about the phenomenon which was studied.

## Results and discussion

Findings of this stud are presented with the support of literature to generate meaning which is based on existing relevant sources and previously conducted research studies. Themes and sub-themes that are reflecting IPs of pregnant women are presented with relevant quotations from the participants (Table 1).

**TABLE 1:** Themes and sub-themes reflecting the indigenous practices of pregnant women at the Dilokong hospital in Limpopo province, South Africa.

Themes	Sub-themes
Indigenous practices based on ancestral knowledge	Indigenous practices after conception. Restrictions during pregnancy. Practices aimed at correcting malpractices. Indigenous knowledge transferred from elders to young ones. Outcomes of pregnancy based on one’s beliefs. Significance of indigenous practices.
Indigenous practices based on spiritual diviners versus church principles	Different pregnancy protection practices. Period of protection. Rationale for utilisation of the protection Significance of the practices towards pregnancy outcomes. Right to prescribe the protection.
Restricted practices versus instructions followed during pregnancy and labour	Spiritual results of not honouring instructions. Physical signs and symptoms of not honouring instructions. Peoples’ influence towards the outcomes of the pregnancy.
Indigenous practices during labour and delivery	Procedures executed for prevention of bad spirit. Practices for precipitating delivery process. Consequences of the women not obeying instructions from THPs. Consequences of disobeying instructions related to the infant. Indigenous practice after delivery.

### Theme 1: Indigenous practices based on ancestral knowledge

The study’s findings reflect that the participants had described different practices that they were engaged in related to IPs during pregnancy, labour, and delivery. Six sub-themes emerged under this theme; IPs after conception, restrictions during pregnancy, practices aimed at correcting malpractices, indigenous knowledge transferred from elders to young ones, outcomes of pregnancy based on one’s beliefs, and the significance of IPs.

#### Sub-theme 1.1: Indigenous practice after conception

The findings of this study reveal practices which the pregnant women engage in immediately after conception. A story of indigenous practice after conception was indicated by a participant who said:

’[*O*]nce you become aware that you are pregnant, they will prepare for you for some solemn obligatory prescription (ditaelo) like Joko tea which is very weak so as to protect you from evil spirits and witchcraft.’

Furthermore, a participant indicated:

’Yes, that you will always live with it and drink it to protect one to have abortion because of people who might be jealous*.*’

Beliefs in witchcraft are a contributory factor to the delay in the first attendance at the ANC as pregnant women are visiting the THPs immediately when they discover that they are pregnant (Banda 2013:7).

#### Sub-theme 1.2: Restrictions during pregnancy

The study’s findings reveal the restrictions that pregnant women have to adhere to during pregnancy as indicated by the participants in their own words:

’Then, if you are pregnant and someone who is also pregnant but low with gestational age, shares drinking water with you, you wait for her to bath, so that you will be able to walk across her soiled used water to get rid of the consequences, like she denies helping me get rid of the consequences (makgoma) but you know her.’

Peltzer et al. (2009:159) confirm that traditional healers are usually the first professionals consulted by people with health problems and the healers will tell them about the restrictions during pregnancy, as they are more accessible geographically and providing a culturally accepted treatment.

#### Sub-theme 1.3: Practices aimed at correcting malpractices

The study’s findings reveal that there are practices which are aimed at correcting malpractices during pregnancy. The findings are supported by the participant who indicated:

’The egg of the killed hen will be taken and will break it and split it on the middle of my head, and this will weakens the magic then I will be released.’

In support of the study’s findings, Truter (2007:59) indicates that traditional medicine is the most common therapeutic method used by African traditional healers for protecting patients from any form of possible afflictions, such as miscarrying or birth complications.

#### Sub-theme 1.4: Indigenous knowledge transferred from elders to young ones

The findings of the study indicate that the indigenous knowledge is transferred from elders to the young. It was indicated by a participant who said:

’This is the information I have learnt from my elders of using some of this medicinal plants to protect my pregnancy.’

And:

’There is a potion that is added into [*sic*] the water which is put aside specifically to be drunk by a pregnant woman, when she needs water. Elders know and have taught us that.’

Many traditional practitioners are mostly elders without education, who have received knowledge of medicinal plants and their effects on the human body from their forefathers (Furber et al. 2009:672).

#### Sub-theme 1.5: Outcomes of pregnancy based on one’s beliefs

The study’s findings reveal that the participants displayed beliefs related to the outcomes of pregnancy. A positive belief was indicated by the participant who said:

’It depends on the beliefs of the pregnant woman. If she does not believe in indigenous practices, the child will be born a healthy baby. It will just be normal before and after birth. Some women believe that these rituals are genuine and indispensable that of course is helpful. Once they arrive at the hospital they bear their children with less labour.’

Papen (2008:386) states that IPs take shape around the cultural traits that are passed from one generation to the next. These practices are deeply rooted and embedded in these societies and, therefore, they become part of the people’s lifestyle. They are innate to such an extent that it is difficult to try and change these beliefs and practices, since people have adhered to them throughout their entire lives. Believe systems play a major role in the health-seeking behaviour of individuals (Shaikh & Hatcher 2005:52).

#### Sub-theme 1.6: Significance of indigenous practices

The study’s findings indicate that IPs are significant to individuals which was confirmed by the participant who stated:

’Mmm! They also give us cords, which are white on which three small pieces of blue cloths are sewn, to protect from miscarriages.’

Another participant added:

’Mm! when you are eight to nine months they will give you a boiled Klim which will protect you against witches, enemies, the people who want to bewitch you by taking your urine cannot be successful because you will be passing milk only.’

These findings were supported by the study conducted by Truter (2007:58) stating that herbal medication is the most common therapeutic method used by the African traditional healers for protecting patients from possible afflictions. Therefore, they prepare powders and earth ointments which comprise animal fat, clay, and ashes for their patients.

### Theme 2: Indigenous practices based on spiritual diviners versus church principles

The study’s findings reflect IPs which were based on the anomaly between spiritual diviners and church principles. Five sub-themes emerged under this theme, namely, different pregnancy protection practices, period of protection, rationale for utilisation of protection, significance of the practices towards pregnancy outcome, and rights to prescribe the protection.

#### Sub-theme 2.1: Different pregnancy protection practices

The findings of the study reveal that there are different pregnancy protection practices. It was indicated by a participant who said:

’At our church, we use these solemn prescriptions whether pregnant or not, but when you are pregnant we use these to protect us from curses or witchcraft, so that witches cannot bewitch you on the day of giving birth.’

Another participant indicated:

’[*S*]olemn tea, solemn Vaseline, salt, coffee, and all are solemnised at church by special people, with light tea we use to bath and drink, then we use salt after usual bath you use water which was not used then you draw in some salt and bath with solemn salt. We put fine particles of it in your shoes before putting them on.’

These findings are supported by the study conducted by Peltzer et al. (2009:158) that confirms that in the Eastern Cape, the THPs treatments prior to delivery vary from rubbing, medicine for bathing, ingestion, or referral to the traditional birth attendant for assistance.

#### Sub-theme 2.2: Period of protection

The study reveals that the THPs suggest a period of protection for their clients during pregnancy as indicated by the participant who said:

’You do not take them off [*strings tied around the abdomen*]. They must be part of your body. You only take them off when you are experiencing labour pains.’

Another participant elaborated:

’What I can tell you is that I attended church and we use what is prescribed by the church for us to protect our pregnancies so that we can reach term.’

The study’s findings are supported by the study conducted by Choudry (1997:537) which confirms that THPs are considered knowledgeable and skilful in maternal care by people in communities when a gestational age is calculated by the position of the moon and the use of *Ritlangi*, which is a thread of grass tied around women’s waists, that provides comprehensive input towards the management of pregnancy and birth.

#### Sub-theme 2.3: Rationale for utilisation of the protection

During data collection, it was found that the participants had knowledge related to important reasons for adhering to their traditional medicine in relation to their conditions. A participant said:

’ [*S*]o that you will not be delayed when you give birth to the baby. It also makes the process of giving birth less painful.’

Another participant said:

’It depends on the neighbours, whether they are enemies for conjuring the demons to the pregnancy, can make it difficult for you to bear the baby normally but through an operation.’

In support of the study’s findings Waiswa et al. (2008:14) state that pregnant women in Uganda consult the traditional birth attendant during ANC because they perceive them as effective caregivers, as they provide herbal medicine to take care of their pregnancies.

#### Sub-theme 2.4: Significance of the practices towards pregnancy outcomes

The findings of the study suggest that practices have significance towards pregnancy outcomes. It was evidenced by the response of the participant who said:

’In my first pregnancy, I consulted with the soothsayer of the St John Apostolic Church and she gave me some cords to use. Because I didn’t like the cords, I used them for a short time and buried them among the underwear I seldom use. The cord had a knot which I was ordered to undo or unfasten when I was about to give birth, I could not unfasten the knot because I forgot everything. I did not remember that I had the cord.’

Lau (2012:29) indicated that knowledge should only be considered if it does not hold any harm to pregnancy. Lau further stated that considering traditional practices for pregnant women might reduce the burden on public hospitals, allowing time and resources to be spent on problematic pregnancies when the practices are safe.

#### Sub-theme 2.5: Rights to prescribe the protection

The study’s findings reflect that the rights to prescribe the protection rest with prophets in the church. That was indicated by the participant who said:

’Yes, you can go to the prophet. The prophet will prescribe (ditaelo) like tea from Moria with picture of our Bishop.’

Another participant added:

’I am a member of the Zion Christian Church, but the elders tell us these cultural practices. Sometimes, the solemn prescriptions of churches surpassed by the power of the curses, then we consult with traditional healer so that they can give us the potions.’

Participants maintained that, although some traditional birth attendants could not read or write, they had a sound knowledge of attending to birth. It was further stated that pregnant women relied on traditional birth attendants for management of pregnancy, based on the attendants’ expert knowledge (Mulaudzi & Ngomane 2003:23).

### Theme 3: Restricted practices versus instructions followed during pregnancy and labour

The findings of the study revealed that there are restricted practices versus instructions followed during pregnancy and labour where women experience pregnancy-related restrictions which are accepted as norms in their cultures. The sub-themes which have emerged under this theme which confirmed the practices, namely, spiritual consequences of not honouring instructions, physical signs and symptoms of not honouring instructions, and people’s influence towards the outcomes of the pregnancy.

### Sub-theme 3.1: Spiritual consequences of not honouring instructions

The study’s findings reflect that some participants do not honour the instructions of spiritual practices as indicated by the participant who said:

’Tremendously! After I returned to the soothsayer to report what I had experienced, she opened the Bible and it was revealed to her that I did not unfasten the knot. It was the time that I remember I hid the cord among ’panties’ which I do not wear when I am pregnant.’

Moloi (2005:118) stated that spiritually ancestors must be respected and honour their instructions because it is believed that they are part of their people.

#### Sub-theme 3.2: Physical signs and symptoms of not honouring instructions

The study findings reveal that participants had knowledge about physical signs and symptoms of not honouring instructions as verbalised by one of participant who said:

’Yes, I was seven months pregnant; now I experienced a serious water-like discharge.’

Another participant said:

’A pregnant woman will experience the taboo related diseases and physical discomfort generally as makgoma; examples of disease are swelling abdomen and flatulence. Sometimes, a miscarriage is possible.’

In biomedical science, the above precautions are not applicable because they are referred to as myths and they cannot be proved to be true or right. However, non-compliance with the ancestral instructions might result in individuals experiencing bad luck (Moloi 2005:121).

#### Sub-theme 3.3: People’s influence towards the outcomes of pregnancy

The study reveals that outsiders can influence pregnancy outcomes as indicated by one participant who said:

’Evil people draw on the roads and if you can tread on them, you are cursed. If you put solemn salt in your shoes and used Vaseline, their curse is but nothing.’

In support of this sub-theme, Mathole et al. (2004:129) indicate that women in Zimbabwe feel that pregnancy has to be kept secret during the early stages for fear of witchcraft. The women further state that the pregnancy is protected from evil spirits that may be inflicted by jealous people who would bewitch the pregnant mother to give birth to a malformed infant or to have a miscarriage.

### Theme 4: Indigenous practices during labour and delivery

The study reveals that the participants displayed engagement with IPs during labour and delivery. Five sub-themes emerged from this theme, namely, procedures executed for prevention of bad spirits, practices for precipitated delivery process, consequences of the woman not obeying instructions, consequences of disobeying instructions related to the infant and IPs after delivery.

#### Sub-theme 4.1: Procedure executed for prevention of bad spirits

The study reveals that participants believed that indigenous procedures executed during labour and delivery prevented bad spirits. One participant indicated:

’Yes, if the evil acts are strongly done to you and if you’ve got hairs on your head, the egg yolk will not flow down your head.’

Another participant stated:

’Then, they will take that killed hen, burn its legs to ashes, then I eat that powder and, therefore, drink warm water, then I will be released.’

Maimbolwa et al. (2003) state that traditional birth attendants advise pregnant women to use traditional medicine as a way of preserving pregnancy and chasing away bad spirits that can affect the pregnancy. Maimbolwa et al. (2003) further stated that participants show trust and faith in what their carers advise them to do during pregnancy.

#### Sub-theme 4.2: Practices for precipitated delivery process

The findings of the study indicate that pregnant women are given traditional medicine to assist with quickening the labour process. This was confirmed by the participant who said:

’They will take that killed hen, burns its legs to ashes, then I eat that powder and, therefore, drink warm water, then I will be released.’

Another participant indicated:

’He instructed his wife to give me a certain potion which smells like the goat dumps in water; yes indeed you cannot ask what the potion is made up of.’

Choudry (1997:537) indicates that traditional birth attendants are considered knowledgeable and skilful in maternal care; roots of Xirhakarhani (a traditional analgesic) are boiled and the water mixture is given to the woman in labour to drink to relieve excessive labour pains.

#### Sub-theme 4.3: Consequences of women not obeying instructions from THPs

The findings of the study indicate that there were instructions that the participants did not obey which led to negative results. That was confirmed by one of the participant who said:

’I delivered my baby with an operation’, and, ’Yes, particularly when the doctor told me that the baby cannot be born normally and so I have to go to operation. The demons were conjured into me and managed to delay the process of delivery because I didn’t follow instructions from [*the*] TH’.

Another consequence was indicated by a participant who said:

’Her pregnancy duration may be extended; she may have miscarriage especially if the solemn cord was not used according to the beliefs of the Zion Christian Church.’

It is believed that non-compliance with a rule might result in difficulties during delivery. Pregnant women should take responsibility for their health by reporting earlier to health care providers for the prevention of complications (Lau 2012:31).

#### Sub-theme 4.4: Consequences of disobeying indigenous instructions related to the infant

The study’s findings reveal that the participants who did not obey the instructions related to IPs experienced bad consequences in relation to their new born infants. A participant verbalised this by saying:

’Sometimes, the baby is abnormal and present with disability of some kind, mmm! Or madness.’

Another indicated that:

’You can either deliver prematurely, or lose the baby in the form of miscarriage, or sometimes it happens that few days after the baby is born, she/he dies.’

These findings are supported by Camacho, Castro and Kaufman (2006:359) who indicated that when indigenous instructions are not obeyed, unfavourable perinatal and neonatal health problems occur to newborn infants. Camacho et al. (2006:359) believe that non-compliance with indigenous rules might result in difficult delivery or abortion, and further state that in communities, the consequences of these imbalances are specialist intervention to restore the balance by using herbs or rituals, depending on the causes that have created the problem.

#### Sub-theme 4.5: Indigenous practices after delivery

The findings in the study reveal that participants displayed beliefs related to IPs after delivery as verbalised by one of the participants who said:

’There are some traditional treatments that are applied on the new born babies, for the baby to become strong quickly, the elders smear Dupa which is a muti used to treat childhood illness, is applied on the skin of the monkey, and burn it in a closed room so that the baby can inhale the smoke.’

Another participant indicated:

’And also the Vaseline, Vicks, and cords (motlemo). We mix Vaseline and Vicks and apply it on the entire body and you will also use another prescription of solemn water with a bit of sand in it, which has been prayed for. After you have taken the usual bath with soap, then you drop of solemn water in the unused water then you bath.’

Gracey and King (2009:71) indicate that perinatal and neonatal health outcomes ’ including deaths ’ are pressing issues, especially in developing countries. Several interventions could cost-effectively save many of the lives of the infants. These interventions include improved clinical care to poorly served groups, engaging families and communities, and improving home-care practices.

## Conclusion

The study’s findings indicate that IPs are regarded as an honourable health intervention by THPs, families, and pregnant women. The women show trust in methods used to preserve pregnancy, labour, and delivery. IPs by pregnant women still continue and, like cords around their waists, are still observed during physical examinations. However, there is a reduction of prescribed potions mixed with cooldrinks for use to accelerate labour and to prevent negative consequences, because the potential toxicity has been explained during the provision of health education. These findings call for health care professionals to emphasise training of and workshops for the THPs for effective implementation of IPs to enhance improvement of negative consequences during pregnancy, labour and delivery.

## Recommendations

Communication and understanding of cultural practices by health care providers and THPs would increase knowledge of earlier attendance of pregnant women at the health facilities. South Africans should have global knowledge of, as well as respect for and accept the function of IPs as cultural beliefs. Health care professionals should respect and protect IPs by formulating programmes for ubuntu meetings with the THPs.

Training workshops and traditional awareness campaigns for THPs, church leaders, elders, and communities must be conducted to strengthen community involvement and active participation in issues pertaining to ANC. Health care professionals must collaborate with conventional clinical services during community-based programmes to minimise the negative effects. Policymakers should develop strategies to protect women by the intervention of specialists in restoring the balance between using prescriptions, religious rituals and biomedicine. Educational sessions during clinical service delivery should be provided, particularly during the physical examination of pregnant women for the promotion of a healthy life style. Churches should also promote change in food habits. Food supplements need to be provided in areas where a deterioration in the nutritional status of individuals is apparent. The council of the spiritual diviners and church leaders should be advised to formulate rules in collaboration with health professionals to ensure that the health and well-being of pregnant women and the unborn baby is ensured.
